# An intergenerational study of perceptions of changes in active free play among families from rural areas of Western Canada

**DOI:** 10.1186/s12889-016-3490-2

**Published:** 2016-08-19

**Authors:** Nicholas L. Holt, Kacey C. Neely, John C. Spence, Valerie Carson, Shannon R. Pynn, Kassi A. Boyd, Meghan Ingstrup, Zac Robinson

**Affiliations:** Faculty of Physical Education and Recreation, University of Alberta, Edmonton, AB T6G 2H9 Canada

**Keywords:** Play, Children, Youth, Rural, Ecological, Community, Qualitative, Physical activity sport

## Abstract

**Background:**

Children’s engagement in active free play has declined across recent generations. Therefore, the purpose of this study was to examine perceptions of intergenerational changes in active free play among families from rural areas. We addressed two research questions: (1) How has active free play changed across three generations? (2) What suggestions do participants have for reviving active free play?

**Methods:**

Data were collected via 49 individual interviews with members of 16 families (15 grandparents, 16 parents, and 18 children) residing in rural areas/small towns in the Province of Alberta (Canada). Interview recordings were transcribed verbatim and subjected to thematic analysis guided by an ecological framework of active free play.

**Results:**

Factors that depicted the changing nature of active free play were coded in the themes of less imagination/more technology, safety concerns, surveillance, other children to play with, purposeful physical activity, play spaces/organized activities, and the good parenting ideal. Suggestions for reviving active free play were coded in the themes of enhance facilities to keep kids entertained, provide more opportunities for supervised play, create more community events, and decrease use of technology.

**Conclusions:**

These results reinforce the need to consider multiple levels of social ecology in the study of active free play, and highlight the importance of community-based initiatives to revive active free play in ways that are consistent with contemporary notions of good parenting.

## Background

Physical activity is associated with healthy body weights, along with musculoskeletal, cardiovascular, neuromuscular, cognitive, and social development in children and adolescents [1, 2]. Yet, for example, only 4 % of girls and 9 % of boys meet Canadian Physical Activity Guidelines (i.e., one hour of daily moderate-to-vigorous physical activity) [3]. One of the major reasons for low overall physical activity levels is the decline of active free play (AFP), a trend observed in numerous countries [4–10]. AFP is a specific type of play, defined as spontaneous and voluntary activities that take place outdoors with minimal or no adult control [11]. Findings from systematic reviews consistently demonstrate the time children spend playing outdoors correlates positively with their physical activity [12–15]. Access to AFP outdoors is also essential for healthy child development [16].

Parents are the most frequently reported barrier to children’s engagement in AFP due to their concerns about traffic safety and ‘stranger danger’ [4, 17–23]. Other barriers to AFP include the presence of teenagers in play areas, access to play areas, playground maintenance, and neighborhood design [11]. Weather may also influence AFP. For instance, children aged 8–12 years-old in Northern climates tend to engage in more overall physical activity in spring/summer compared to fall/winter [24]. Several studies examining AFP have interviewed children [17, 20, 25] and parents [26–28] to identify barriers and facilitators of AFP. Another way to understand factors that influence children’s engagement in AFP is to examine how it has changed over time among multiple generations of families, including grandparents [11].

Previous intergenerational studies of AFP – conducted in urban settings – have revealed that time spent outdoors has declined, children face more parental rules and restrictions than in the past, and they are more likely to participate in structured, supervised, and indoor activities [10, 29, 30]. Social changes have also influenced changes in AFP. For example, a study conducted in New York City showed that children’s access to public play spaces declined from the 1930s to the early 2000s due to public divestment in neighborhood parks and playgrounds, violence in these spaces, and the commercialization and privatization of play activities [31]. A study with children and parents from suburban areas of Newcastle, Australia, demonstrated that an increase of home-based leisure technology and parental restrictions limited where children might play [32].

More recently, a project in the city of Auckland, New Zealand [10, 33] examined intergenerational changes in play through focus groups with 68 parents. There was near universal agreement that children had less freedom to explore neighborhoods than their parents enjoyed, spent more time being driven in vehicles, and more time being sedentary in the ‘electronic bedroom.’ Children were subjected to close monitoring and surveillance due to their parents’ perceived safety concerns, and parents used spatial and temporal boundaries to limit where their children went and for how long. Yet, while parents’ memories of their own childhood were rather nostalgic, they also recalled working from an early age and receiving harsh discipline, which often limited time for play. Furthermore, while contemporary technology may have increased indoor sedentary behaviors, some sedentary behaviors (e.g., television, board games, cards) have long featured in children’s lives [34]. Though there is a growing body of evidence about how AFP has changed in urban areas, there is little understanding of how it has changed across generations in rural settings [11].

Children in rural settings may have more opportunities to play in outdoor environments [35], yet they also face barriers to engaging in AFP that differ from their urban counterparts [36]. For example, low residential density may reduce children’s opportunities for spontaneous engagement in AFP [37]. The rural childhood idyll (i.e., the romantic notion that rural areas are a place of innocence, play, adventure and companionship featuring freedom and the absence of adult surveillance) has also been questioned. Researchers have shown that children in rural areas are often under the gaze of adults, lack recreational space, and are restricted by their parents [8, 36, 38, 39].

As noted earlier, the majority of AFP research - and previous intergenerational studies - have been conducted in urban areas [4, 11]. Furthermore, there are several limitations of previous intergenerational research on AFP. For instance, studies have relied on parents’ accounts [39] or examined changing attitudes between parents and their children [40], but rarely have the views of grandparents’ been included [11]. Results of some previous studies may have been influenced by family mobility. For instance, many parents in Witten and colleagues’ [10] intergenerational study grew up in rural settings and then moved to urban and suburban areas of Auckland. It is possible that some of the intergenerational changes reported by Witten et al. reflected differences in rural versus urban childhoods rather than changes in AFP per se. Hence, the purpose of this study was to examine perceptions of intergenerational changes in AFP among families from rural areas. More specifically, we addressed two research questions: (1) How has AFP changed across three generations? (2) What suggestions do participants have for reviving AFP?

### Ecological framework of active free play

This study was guided by a framework depicting factors that influence children’s engagement in AFP at various levels of social ecology [11]. The AFP-specific social-ecological framework is organized from more proximal to more distal issues, beginning at the child level (e.g., child age, competence, and gender) and moving to parental restrictions (safety concerns and surveillance), neighborhood and physical environment (fewer children to play with, differences in preferences for play spaces between parents and children, accessibility and proximity, and maintenance), social changes (reduced sense of community, changing good parenting ideal, changing roles of parents, privatization of playtime and play spaces), and policy issues (need to give children voice). These ecological systems are also influenced by historical developments over time. Thus, the use of this ecological framework facilitated the analysis of more proximal issues and how they relate to, and are influenced by, more distal issues over time.

## Method

### Site selection and participant recruitment

To be eligible to participate in this study, families must have (a) included grandparents, parents, and children, (b) included children aged 7–12 years old, and (c) been born and raised, and currently resided in, the same small town/rural area in Alberta (Canada). Four small town/rural areas were identified based on Statistics Canada criteria [41]. Recruitment flyers were posted in community centres and local newspapers. Snowball sampling was also used. Potential participants contacted/were contacted by a member of the research team, their eligibility to participate in this study was established, and interviews were arranged. Institutional research ethics board approval was obtained. Grandparents and parents provided written informed consent. Parents provided written informed consent for their children. Children provided verbal assent prior to their interview.

### Participants

Forty-nine people from 16 families participated in this study. There were 15 grandparents (14 female, one male; *Mean* age = 62.6 years, *SD* = 2.1 years, range = 58–67 years) – in one family grandparents were unavailable for an interview, 16 parents (15 female, one male; *Mean* age = 36.7 years, *SD* = 5.2 years, range = 28–49 years), and 18 children (nine male, nine female; *Mean* age = 9.1 years, *SD* = 2.0 years, range = 7–12 years) – in two families two children within the specified age range were interviewed at the parents’ request. All participants were Caucasian with European heritage and all adults described themselves as being middle income, reflecting demographics typical of the areas we studied.

### Data collection

Each participant completed one individual semi-structured interview that was conducted in a quiet area of the family home free from distractions. Typically, one researcher interviewed the grandparent while the other researcher interviewed the parent then the child. Interviews with parents and grandparents lasted approximately 45–60 min, and interviews with children lasted approximately 20 min. An interview guide was developed and adapted for each generation. For example, grandparents were asked a series of questions about their own childhood play, their children’s, and their grandchildren’s play. Parents and grandparents were also asked questions about their own involvement in the children’s (or grandchildren’s) play, along with perceived barriers and facilitators of play. Next, they were asked a series of questions about how play has changed across the generations of their family. In the concluding part of the interview, all participants were asked to provide suggestions regarding how to revive AFP.

### Data analysis

Audio files were transcribed verbatim and participants were assigned a code (e.g., grandparent 1 = GP1, parent 1 = P1, and child 1 = C1). Data were thematically analyzed using an approach that involves comprehending, synthesizing, theorizing, and recontextualizing [42, 43], which has been used in previous work examining AFP [44]. Two analytic strategies were used for the initial comprehending step. First, transcripts from each family unit were examined together (i.e., within-case analysis), and an overview of how AFP had changed within each family was written. This step provided context for the next part of the analysis, whereby transcripts were analyzed by generation (i.e., between-case analysis), starting with the grandparents’ data. For example, each grandparent interview transcript was analyzed to identify themes and patterns in the grandparents’ data. This process was then repeated for the parents’ and children’s interview transcripts. The constant comparative technique was used [45]. This involved re-reading data in each theme to ensure they ‘fit.’

The next step was synthesizing. Specifically, we identified the common themes across the grandparents’, parents’, and children’s data that depicted intergenerational changes over time and suggestions for improving AFP. Data from each group of participants were placed in these themes, and a narrative overview of the changing nature of AFP across the generations (and suggestions for improving AFP) was written. During the process of theorizing, we used the ecological framework of AFP [11] to organize and interpret data, particularly in terms of proximal to more distal and chronological factors. A data matrix [46] was created to provide an overview of how AFP changed across generations with respect to the themes identified (Table 1). Finally, the findings were recontextualized through drafting and re-drafting the written narrative.

**Table 1:** Changes in the nature of play

Ecological level	Theme	Grandparents’ era	Parents’ era	Children’s era
Child level	Less Imagination, More Technology	Kids’ games and using imagination	Some kids’ games and imagination, some technology	Few kids’ games, less imagination, more technology
Parent level	Safety Concerns	Few safety concerns	Some safety concerns	Extensive safety concerns
	Surveillance	Few parental restrictions on play	Some parental restrictions on play with little monitoring	Extensive parental restrictions on play and compulsory monitoring
Community level	Other Children to Play With	Few friends, but good friends	More friends living closer	Few friends to play with
		Extended family important	Extended family still important	Few extended family members close
	Purposeful Physical Activity	Walking/riding to meet friends out of necessity	Sometimes out of necessity, sometimes for recreation	Rare. Not out of necessity
	Play Spaces and Organized Activities	Few formal play spaces, no organized activities	More play spaces, some organized activities	Lots of play spaces and facilities, lots of organized activities
Social changes	Good Parenting Ideal	Virtually no parental involvement in play	Some parental involvement in play	Parental involvement in play expected and needed, but parents too busy working.

### Methodological rigour

Strategies were embedded in the research design to help establish methodological rigour [47]. The site selection and sampling criteria enabled us to recruit participants who could provide ‘information-rich’ accounts [43]. Data collection and analysis occurred in three phases, which allowed for self-correction during the study process and enabled us to make judgments about data saturation. Obtaining data from at least three members of each family unit provided opportunities for data triangulation. The analytic steps were led by the first author and scrutinized by other members of the research team. This team approach during analysis helped provide analytic balance [48].

## Results

### The changing nature of active free play in rural areas

#### Child level

##### Less imagination, more technology

Grandparents recalled playing traditional children’s games and using their imagination to create games using materials they found nearby. For example, GP8 said, “There were always things to do…. you know, do our own thing. I think we were much better at entertaining ourselves.” Parents also played traditional children’s games and used their imaginations, but technology was becoming a feature of their play. For instance, P12 said, “I remember getting the first *Nintendo* when we were little, so we always had computers when we were inside.” Children did not play traditional kids’ games much, and used their imagination to create games less than grandparents or parents. In fact, when asked about what they play, almost every child mentioned some form of electronic games. C5 said:

I like to play videogames. [Children] can’t keep their eyes off the games. My friend, he can’t keep his eyes off his videogames, he never goes outside. He just plays his videogames. That’s what I feel like doing when I’m a teenager.

#### Parent level

##### Safety concerns

Grandparents and parents had very few safety concerns during their childhood. For instance, P14 recalled where she grew up was “quiet, you know, not a lot of traffic, not a lot of people really necessarily around, so you had the freedom of going out into the street and playing if you wanted to.” In stark contrast, safety was a major barrier for the children’s play. For instance, when asked about safety, C13B said:

Being out in town and not having anybody supervising you that’s an adult or older than you. It’s kinda hard too to say that it’s safe because there’s many things that happen. I would probably say the most safe place would be here at home, not at the park or out of the yard.

##### Surveillance

As safety concerns were not a feature of the grandparents’ play, there were very few parental restrictions or surveillance strategies during this era. Such restrictions began to emerge in the parents’ era and became increasingly prevalent during the children’s era. For instance, P3 remembered that as a child he had “some parameters, you could only go this far… With my kids, it’s different. They can’t just go and play ‘cause the park isn’t close enough and I can’t see them.” Similarly, GP4 said, “people are much more hesitant to ever let their kids out of sight you know.” Parents used several types of surveillance, including check-in times and technology. P3 explained her daughter “has a cell phone, so I always feel safe that I can get a hold of them.” Children also reported several types of parental rules and restrictions, such as not talking to strangers (C7), not leaving the yard (C9), looking out for wildlife (C10), having check-in times (C11A), and not going too far away unless with an adult (C11B).

#### Community level

##### Other children to play with

Grandparents spoke of having few friends who lived close by and the importance of playing with extended family members. GP3 recalled, “we played ball if we could ever find enough people to play [but] my closest cousin or neighbor was a mile and a half away.” For parents, friends often still lived some distance away, but they had more friends to play with outside of the immediate family than during the grandparents’ era. It was different for the children. They needed friends to play with, and lack of friends to play with was a barrier. GP4 said her grandchildren were “probably isolated a little bit… not having friends. Similarly, P4 explained that:

When you used to play in your neighborhood, everybody was outside. You just ran outside and everyone was there… It’s not like that anymore. You don’t see the neighborhood full of kids out there, so it’s harder to go find your friends to hang out with.

##### Purposeful physical activity

Grandparents often engaged in physical activity out of necessity (i.e., active transportation). GP11 said, “Well you know your closest friend would be half a mile away [so] I suppose you’d walk. My mother didn’t drive so you would walk to go visit them and then as you got older you had bicycles.” In contrast to grandparents, parents were more likely to engage in physical activity for recreational purposes. For instance, P3 remembered “I’d ride my bike around the block and to the corner and back. There was a stop sign so we’d ride to the stop sign and back all the time.” Children rarely engaged in active transportation (they would be driven) and their activities like bike riding were almost entirely for recreation. For instance, C1 said, “I ride my bike with her [sister], or have scooter races with her. The scooter races and riding our bikes, we do it on the road.”

##### Play spaces and organized activities

Grandparents had very few spaces for play. As GP2 observed, there were “no organized parks like there are now,” and GP10 said, “I don’t ever recall a playground.” In terms of organized activities, GP4 recalled that “we never did any sports” and GP3 said “I never played organized [sports].” Parents had more resources and spaces to support play than the grandparents and were also more involved in organized sports and other activities (e.g., after-school clubs) compared to the grandparents. Children had lots of places to play. P14 said, “there’s a bazillion parks and if I choose to bring my kid to parks or if I want my kid involved in activities, I know there’s a lot of opportunity for it.” P6 went further and thought that, “kids are just handed everything.” Additionally, the extent to which children were involved in organized activities restricted their engagement in play. P13 captured this succinctly, saying, “organized sports have killed active play.”

#### Social changes

##### Good parenting ideal

The notion of good parenting, in relation to AFP, changed across the generations. The ideas in this theme reflect how parenting around AFP is influenced by broader social changes. For instance, there was virtually no parental involvement in the play of the grandparents because their parents were not expected to be involved in play. But, as GP11 remarked, in her own family there is “definitely more play family-wise. We certainly did things I would not have done things with my parents.” In fact, being involved in children’s play was seen as a feature of good parenting in contemporary society. P15 said “being a good parent is spending time, like quality time with your kids, teaching them new things, having fun together” and P10 said “I think a good parent is involved in both their education and their playing… Playing with them, doing things that they like to do.” Children also highlighted the importance of parents playing with them. C15 said parents should “help them [children] with something or go play with them. C1 said it was important for parents to teach children “how to bounce a ball” and for children to “play pass with their parents.”

However, parents were busy and this restricted the extent to which they could be involved in their children’s play. As P14 said, “some parents just have too much on their plate… there’s no time for free play.” Similarly, GP6 remarked that “The availability of the parents, and, and in this day and age most parents work [and] both parents work. By the time they get home from work they’re done. The last thing they wanna do is go out and play.” Hence, although many parents suggested playing with their children was a feature of good parenting, shifting social structures (e.g., the need to be involved in and/or supervise AFP, along with both parents working) often did not allow for parental involvement in AFP.

#### Suggestions for improvements

##### Enhance facilities to keep kids entertained

One of the most consistent themes for reviving play was to improve facilities (rather than build new facilities). For instance, some parents suggested the need to “upgrade the parks to being better quality” (P1) or to “make the parks more appealing… new equipment with brighter colors” (P3). Another idea was to make playgrounds more appropriate for children of different ages. P6 highlighted that “parks are more geared for little kids” and there is a need for “different types of parks for kids [at different ages] to suit their interests.” Similarly, children talked about improving play spaces. C2 suggested “they [local authorities] could make all the parks newer and stuff and get more ramps for the skate park.” C3 said “We need to have more things to keep kids entertained.”

##### More opportunities for supervised play

Grandparents and parents provided a variety of suggestions for creating opportunities for supervised play, largely in response to overcoming concerns about safety. These suggestions included the creation (or continued use) of supervised programs during the summer. P1 described a program in her town run by two university students:

… at various parks throughout the summer months, and it’s to bring back the old way of play, like kick the can, and skipping rope, and kick the ball, and kids can go play with them at the park… It’s neat to see because it’s the old way of playing.

Similarly, P4 wanted to see similar types of programs near her home, and said there should be “community watches or, you know, community centers where kids can go hang out and play, where there is supervised play.”

##### More community events

Participants suggested that play could be revived by improving a sense of community. P10 spoke enthusiastically about a new community group near her home and the creation of a winter festival, which got children outdoors playing with other children. C3 also suggested the need for community events, saying:

We could have big things happening, like maybe a sponge fight or around like a full thing with the town for you to sign up and then can [play]. Or like a big town scavenger hunt that would go a little bit out of town, like in the county but not that far out. You wouldn’t be that bored.

Offering similar ideas, GP9 told us “I do think there’s areas where people don’t know anybody, where they kind of keep to themselves. [So we did] a scavenger hunt. They like that. Once or twice a year… and the kids love it.”

##### Decrease use of technology

The majority of grandparents, parents, and even children suggested that the best way to revive free play was to decrease the use of technology. Suggestions for decreasing children’s reliance on technology included setting time limits on the use of devices (GP13), monitoring and restricting internet activities (GP14), shutting power off (GP16), putting up an “internet blocker” (GP3), and increasing parental involvement to encourage non-technologically reliant forms of play (GP9). As to the latter point, GP9 said parents should spend more time with their children and engage with them, rather than letting them play on electronic devices. She said parents should “find out how their day was. Ask ‘what would you like to do?’ Just stretch them, rather than say ‘here, play [with this device].’” Some of the children agreed. C16 suggested that parents should “give away some of their video games” to get children to play outside more because, as C3 observed, “when you’re playing on it [video game] you’re not gonna think ‘oh I’m gonna go outside now.’”

## Discussion

Results revealed intergenerational changes in AFP among families from rural areas and suggestions for reviving AFP. Some of the reported changes were consistent with the results of previous intergenerational studies conducted in *urban* areas; perceptions of safety concerns, parental surveillance, and use of technology increased across the generations, while engagement in active transportation decreased [10, 29, 30, 40]. Our findings show that these issues (and others) that may traditionally be considered ‘urban problems’ were prevalent in rural areas. The findings also highlight connections between parenting activities and social changes, and provide some useful suggestions for reviving AFP.

Many of the themes depicting the changing nature of play hinged around the role of parents – both in terms of specific parenting behaviors and how parenting was influenced by changing social norms. For the grandparents, there was virtually no parental involvement in their play, while parental involvement was expected and needed for the children. Yet, parental involvement in the children’s play was restricted because parents were busy, and often both parents worked. The central idea here is that the ‘good parenting ideal’ (i.e., how parents understand societal expectations for their parenting) has changed over past decades [11]. Contemporary ‘good parents’ perceived the need to monitor their children at all times, and allowing children to roam free is generally considered to be a feature of ‘poor parenting’ [8, 23, 49, 50]. The notion of good parenting is related to social trends. In contemporary society, increased number of mothers in the workforce and both parents working (in two-parent households) means parents spend less time in the family home. Good parenting may involve, for some, working long hours to provide financially for their families rather that spending unstructured free time together [10, 51].

As a consequence of the evolving expectations about good parenting and increasing safety concerns, children faced far more extensive parental restrictions and surveillance of their play than grandparents or parents. Check-in times and carrying cell phones are previously reported monitoring strategies parents in urban areas use [10, 17, 32]. These strategies may provide parents with an illusion of safety [52] and be features of ‘good parenting’ in modern society because, even when children are allowed to play outdoors, they remain subjected to adult monitoring and surveillance.

Contemporary research (in urban settings) suggests that having friends to play with provides ‘safety in numbers’ [44]. The findings emphasized the importance of having friends, but revealed some subtle changes across the generations. That is, while grandparents had few friends, they reported having ‘good friends’ (or very strong friendships) along with extensive interactions with members of their extended family. Grandparents also engaged in active transportation -- out of necessity due to low residential density -- to maintain these friendships. Like the grandparents, children had few friends, but in contrast they did not use independently travel to reach them. Our findings speak to the erosion of close friendships with other children in a community and with extended family members in contemporary rural societies as active transportation has dwindled.

It was clear that while grandparents had few play spaces and almost no organized activities, these factors changed across generations. Children’s increased involvement in organized activities is almost certainly a consequence of parents’ safety concerns. Organized activities include supervision and therefore, presumably, a sense of perceived safety. Yet, by enrolling their children in private/supervised activities, restricting the time they spend outdoors, and driving them to these activities, parents further reduce the number of children to play with and add to the amount of road traffic. Again, these ‘social traps’ [4] are most likely a reflection of the changing nature of the good parenting ideal.

Reflecting safety concerns and the perceived need for supervision and monitoring, grandparents and parents called for their communities to provide more opportunities for supervised play. An implication here is that local authorities may wish to consider investing resources at the program level to increase AFP. Importantly, as recent research from Norway has shown, such programs should not involve excessive adult planning and involvement in play, but rather provide supervised opportunities for spontaneous and child-initiated play [53]. Participants also called for more events to create a sense of community or social cohesion, which would facilitate AFP. Combined, these ideas reflect the notion of placing more ‘eyes on where children play’ through community mobilization [44].

Finally, grandparents, parents, and children suggested decreasing children’s use of technology would increase AFP. Tremblay and colleagues [16] surmised that when children spent more time in front of screens they are more likely to be exposed to cyber-predators, violence, and eat unhealthy snacks. They questioned whether keeping children indoors (and playing with electronics) is really safer than playing outdoors. Our participants’ common-sense suggestions (e.g., time limits on use of devices, restricting and monitoring internet activities) are valuable and consistent with public health guidelines [54].

The results presented herein portrayed the common themes across families. There were some subtle variations. For instance, while safety was certainly a concern for all families, some parents recognized that saturated and sensationalized traditional and social media coverage of incidents involving children increased their perception of risk rather than actual risk. In terms of suggestions for improvements, not all suggestions were endorsed by all families. For instance, some families focused more on the need for community-building types of activities, while others emphasized the need to reduce use of technology as a key area for improving AFP. These subtle variations highlight that initiatives to revive AFP may require multiple strategies to reach families with different priorities.

Limitations of this study include a reliance on retrospective recall, particularly in terms of grandparents’ and parents’ recollection of their own childhood play. To some extent this was balanced by opportunities to triangulate the participants’ responses. Another limitation was that while we may reasonably assume that grandparents were more active than children, we did not have any objective measures of physical activity. The fact that the sample was dominated by grandmothers and mothers reflects child-rearing responsibilities in the families but restricts analysis of gender differences in relation to AFP. For instance, there may be subtle differences in the ways in which mothers and fathers support AFP that were not identified in the current study. As with most qualitative studies, the generalizability of these findings are limited to people similar to those who participated in this study and to those who live in rural areas. In this respect, it is important to note that the rural areas studied are quite remote due to the vast size of Alberta (the Province covers an area of over 660,000 km^2^) and the relatively small population (just over four million people in 2015), the majority of whom reside in two major cities (Edmonton and Calgary) [55]. Finally, because this study focused on AFP (which, by definition, involves minimal adult supervision) we did not consider how the types of physical activity in which children engage during school hours (e.g., during supervised recesses) has changed, and this remains an avenue for future research.

## Conclusions

Collectively, our results show that what might be considered ‘urban problems’ are also prevalent in rural areas and the changing social forces that influenced AFP. The findings highlight the need to consider multiple levels of social ecology to revive AFP, and point to the particularly important role of community building and community programming to create (or directly provide) the sense of supervision that is essential to modern conceptions of good parenting. More specifically, implications for policy makers (e.g., local municipalities) and professionals (e.g., recreation programmers) in rural areas include maintaining and/or renovating play areas to provide AFP opportunities for children of different ages, providing supervised opportunities for spontaneous and child-initiated play, and creating community-building events to support AFP. From a public health perspective, initiatives to promote AFP must be aligned with modern conceptions of good parenting and our study would suggest that merely encouraging parents to let their children play outside unsupervised – a strategy used in some public health campaigns [56] – is unlikely to be successful because it does not appeal to the conception of what it means to be a good parent.

## Abbreviations

AFP: Active Free Play

## References

[1] Janssen I (2007). Physical activity guidelines for children and youth. App Phys Nutr Metabolism.

[2] Janssen I, Leblanc AG (2010). Systematic review of the health benefits of physical activity and fitness in school-aged children and youth. Int J Behav Nutr Phys Act.

[3] Colley RC, Garriguet D, Janssen I, Craig CL, Clark J, Tremblay MS (2011). Physical activity of Canadian adults: accelerometer results from the 2007 to 2009 Canadian Health Measures Survey. Health Rep.

[4] Carver A, Timperio A, Crawford D (2008). Playing it safe: the influence of neighbourhood safety on children’s physical activity - a review. Health & Place.

[5] Fyhri A, Hjorthor R, Mackett RL, Fotel T, Kytta M (2011). Children’s active travel and independent mobility in four countries: Development, social contributing trends, and measures. Transp Policy.

[6] Singer DG, Singer JL, D’Agostino D, DeLong R. Children’s pastimes and play in sixteen nations: Is free play declining? Am J Play. 2009; Winter:283–312.

[7] Sturm R. Childhood obesity - What we can learn from existing data on societal trends, Part 1. Preventing Chronic Disease. 2005;2(1):A12. [serial online]. PMC1323315 15670465

[8] Valentine G, McKendrick J (1997). Children’s outdoor play: Exploring parental concerns about children’s safety and the changing nature of childhood. Geoforum.

[9] Wen LM, Kite J, Merom D, Rissel C (2009). Time spent playing outdoors after school and its relationship with independent mobility: a cross sectional survey of children aged 10–12 years in Sydney, Australia. Int J Behav Nutr Phys Act.

[10] Witten K, Kearns R, Carroll P, Asiasiga L, Tava’e N. New Zealand parents’ understandings of the intergenerational decline in children’s independent outdoor play and active travel. Child Geogr. 2013;11(2):215–229.

[11] Lee H, Tamminen KA, Clark AM, Slater L, Spence JC, Holt NL. A meta-study of qualitative research examining determinants of children's independent active free play. Int J Behav Nutr Phys Act. 2015;12:5. 10.1186/s12966-015-0165-9 PMC4318368 25616690

[12] Ferreira I, Van der Horst K, Wendel-Vos W, Kremers S, Van Lenthe F, Brug J (2006). Environmental correlates of physical activity in youth: a review and update. Obes Rev.

[13] Hinkley T, Crawford D, Salmon J, Okely A, Hesketh K (2008). Preschool children and physical activity: a review of correlates. Am J Prev Med.

[14] Sallis JF, Prochaska JJ, Taylor WC (2000). A review of correlates of physical activity of children and adolescents. Med Sci Sports Exerc.

[15] Sterdt E, Liersch S, Walter U (2014). Correlates of physical activity in children and adolescents: a systematic review of reviews. Health Educ J.

[16] Tremblay MS (2015). Position statement on active outdoor play. Int J Environ Res Public Health.

[17] Brockman R, Fox KR, Jago R (2011). What is the meaning and nature of active play for today’s children in the UK?. Int J Behav Nutr Phys Act.

[18] Burdette HL, Whitaker RC (2005). A national study of neighborhood safety, outdoor play, television viewing, and obesity in preschool children. Pediatrics.

[19] Carver A, Timperio A, Hesketh K, Crawford D (2010). Are children and adolescents less active if parents restrict their physical activity and active transport due to perceived risk?. Soc Sci Med.

[20] Holt NL, Cunningham C, Sehn ZL, Spence JC, Newton AS, Ball GDC (2009). Neighborhood physical activity opportunities for inner-city children and youth. Health Place.

[21] Glenn N, Knight CJ, Holt NL, Spence JC (2013). Meanings of play among children. Childhood.

[22] Timperio A, Crawford D, Telford A, Salmon J (2004). Perceptions about the local neighborhood and walking and cycling among children. Prev Med.

[23] Tranter P, Pawson E (2001). Children’s access to local environments: a case-study of Christchurch, New Zealand. Local Environ.

[24] Carson V, Spence JC (2010). Seasonal variation in physical activity among children and adolescents: A review. Ped Exer Sci.

[25] Veitch J, Salmon J, Ball K (2007). Children’s perceptions of the use of public open spaces for active free-play. Child Geogr.

[26] Dias JJ, Whitaker RC (2013). Black mothers’ perceptions about urban neighborhood safety and outdoor play for their preadolescent daughters. J Health Care Poor Underserved.

[27] O’Brien J, Smith J (2002). Childhood transformed? Risk perceptions and the decline of free play. Br J Occup Ther.

[28] Jago R, Thompson JL, Page AS, Brockman R, Cartwright K, Fox KR (2009). License to be active: parental concerns and 10–11-year-old children's ability to be independently physically active. J Public Health.

[29] Gaster S (1991). Urban children’s access to the neighborhood: changes over three generations. Envir Behav.

[30] Karsten L (2005). It all used to be better? Different generations on continuity and change in urban children’s daily use of space. Child Geogr.

[31] Wridt PJ (2004). An historical analysis of young people’s use of public space, parks, and playgrounds in New York City. Child Youth Environ.

[32] Tandy CA (1999). Children's diminishing play space: a study of intergenerational change in children's use of their neighbourhoods. Aust Geogr Stud.

[33] Oliver M, Witten K, Kearns RA, Mavoa S, Badland HM, Carroll P, Drumheller C (2011). Kids in the city study: research design and methodology. BMC Public Health.

[34] Biddle SJ, Gorely T, Stensel DJ (2004). Health-enhancing physical activity and sedentary behaviour in children and adolescents. J Sports Sci.

[35] Salmon J (2013). Are associations between the perceived home and neighbourhood environment and children’s physical activity moderated by urban/rural location?. Health Place.

[36] Matthews H, Taylor M, Sherwood K, Tucker F, Limb M (2000). Growing-up in the countryside: children and the rural idyll. J Rural Stud.

[37] Walia S, Liepert D (2012). Perceived facilitators and barriers to physical activity for rural youth: an exploratory study using photovoice. Rural Remote Health.

[38] Tucker F, Matthews H (2001). ‘They don’t like girls hanging around there’: conflicts over recreational space in rural Northamptonshire. Area.

[39] Valentine G (1997). A safe place to grow up? Parenting, perceptions of children’s safety and the rural idyll. J Rural Stud.

[40] Karsten L (2003). Children's use of public space: the gendered world of the playground. Childhood.

[41] Statistics Canada. Structure and change in Canada’s rural demography: an update to 2006. Rural and Small Town Canada Analysis Bulletin. 2008; 7(7) http://www.statcan.gc.ca/pub/21-006-x/21-006-x2007007-eng.pdf

[42] Morse JM, Field PA (1995). Qualitative research methods for health professionals.

[43] Mayan MJ (2009). Essentials of qualitative inquiry.

[44] Holt NL, Lee H, Millar CA, Spence JC (2015). ‘Eyes on where children play’: a retrospective study of active free play. Child Geogr.

[45] Glaser BG, Strauss AL (1967). The discovery of grounded theory.

[46] Miles MB, Huberman M (1994). Qualitative data analysis: an expanded sourcebook.

[47] Morse J, Barrett M, Mayan M, Olson K, Spiers J (2002). Verification strategies for establishing reliability and validity in qualitative research. Int J Qual Met.

[48] Patton MQ (2002). Qualitative research & evaluation methods.

[49] Blakely, KS. Parent’s conceptions of social dangers to children in the urban environment. Children’s Environments. Winter. 1994;11:16–25.

[50] Valentine G (1997). “Oh yes I can”. “Oh no you can’t”: children and parents’ understandings of kids’ competence to negotiate public space safely. Antipode.

[51] Kinoshita I (2009). Charting generational differences in conceptions and opportunities for play in a Japanese neighbourhood. J Intergenerational Rel.

[52] Jenkins NE (2006). You can’t wrap them in cotton wool! Constructing risk in young people’s access to outdoor play. Health Risk Soc.

[53] Skar M, Gundersen V, O'Brien L (2016). How to engage children with nature: why not just let them play?. Child Geog.

[54] Tremblay MS, Warburton DER, Janssen I, Patterson DH, Latimer AE, Rhodes RE, Kho ME, Hicks A, LeBlanc AG, Zehr L, Murumets K, Duggan M (2011). New Canadian physical activity guidelines. Appl Physiol Nutr Metab.

[55] Alberta Government. Population. Retrieved from http://economicdashboard.alberta.ca/Population

[56] ParticipACTION. Bring back play. Retrieved from http://www.bringbackplay.ca/

